# Resonance-Based Reflector and Its Application in Unidirectional Antenna with Low-Profile and Broadband Characteristics for Wireless Applications

**DOI:** 10.3390/s16122092

**Published:** 2016-12-09

**Authors:** Lin Peng, Ji-yang Xie, Kai Sun, Xing Jiang, Si-min Li

**Affiliations:** 1 Guangxi Key Laboratory of Wireless Wideband Communication and Signal Processing, Guilin University of Electronic Technology, Guilin 541004, China; mcgrady_guet@163.com (J.X.); 13978385982@163.com (K.S.); jiang_x@guet.edu.cn (X.J.); siminl@guet.edu.cn (S.L.); 2 Key Laboratory of Microwave and Optical Wave-Applied Technology, Guilin University of Electronic Technology, Guilin 541004, China

**Keywords:** resonance based reflector, low profile, broadband, unidirectional, wireless applications

## Abstract

In this research, the novel concept of a resonance-based reflector (RBR) was proposed, and a ring-shaped RBR was utilized to design a unidirectional antenna with low-profile and broadband characteristics. Research found the ring operates as two half-wavelength (*λ*/2) resonators. Then, the resonance effect transforms the reflection phase of the ring RBR, and achieves a reflection phase of 0° < *ϕ* < 180° in a wide frequency range above the resonance. Then, the in-phase reflection characteristic (−90° < *ϕ* < 90°) can be obtained in the wide frequency band by placing an antenna above the RBR with a distance smaller than *λ*/4. Two unidirectional antennas, named Case 1 and Case 2, were designed with the ring-shaped RBRs and bowtie antennas (RBR-BAs). The impedance bandwidths of Case 1 and the Case 2 are 2.04–5.12 GHz (86.3%) and 1.97–5.01 GHz (87.1%), respectively. The front-to-back ratio (FBR, an important parameter to measure the unidirectional radiation) of Case 1 ranges from 5–9.9 dB for frequencies 2.04–2.42 GHz, and the FBR of Case 2 ranges from 5–16 dB for frequencies 2.16–3.15 GHz. The proposed concept of RBR is desirable in wideband unidirectional antenna design, and the designing antennas can be used at the front end of wireless systems—such as indoors communication, remote sensing, and wireless sensor systems—for signal receiving or transmitting.

## 1. Introduction

Broadband antennas have many merits and they have been widely used in radar, mobile communication, remote sensing, and medical examination [1,2,3,4,5]. Requirements for antennas have become more and more restricted with the rapid development of these broadband systems, such as having the characteristics of compact size, low profile, ultrawideband (UWB), and unidirectional pattern. Unfortunately, it is hard to possess all of the above merits in one antenna.

Traditional unidirectional antennas, such as the log-periodic antenna, horn antenna, and Vivaldi antenna [6,7,8], are all wideband and unidirectional. However, the overall sizes of these antennas are usually large and their profiles are high, owing to an end-fire pattern with maximum radiation direction forwarded to one end of the antenna. For example, it has been shown that although there is an impedance bandwidth of 4.5–11 GHz for the log-periodic antenna, the profile is 0.36*λ*, while the area is 0.75*λ* × 0.75*λ* [6]. Impedance bandwidths of 17.4–24 GHz and 1–18 GHz have been achieved for the horn antenna [7] and Vivaldi antenna [8], respectively, however, their profiles are 0.27*λ* and 2.2*λ*, respectively, and their areas are 1.47*λ* × 3.77*λ* and 2.2*λ* × 1.1*λ*, respectively.

The characteristics of unidirectional pattern and low profile are possessed by microstrip antennas [9,10,11]. However, their bandwidths are restricted by the inherent high-quality factor (Q factor, defined by the ratio of energy stored and energy dissipated per cycle).

Planar antennas (PAs) demonstrate very wide bandwidths (for example 3.1–10.6 GHz) and low-profile characteristics, attributed to their open structure and very low Q factor [12,13,14]. However, their radiation patterns are omnidirectional.

One method to transform the omnidirectional pattern of PAs to unidirectional is using a perfect electric conductor (PEC) plane as reflector. This method utilizes the characteristic of a 180° phase transformation of the PEC, therefore, the reflector must be *nλ*/4 (*n* are odd numbers of 1, 3, 5...) away from the radiator to obtain a constructive result for the reflected wave from the reflector and the radiated wave from the radiator [15,16]. In [15], 20.0% bandwidth was achieved with a consequential profile of 0.29*λ* for the antenna. In [16], 23.7% bandwidth was obtained for the antenna with a profile of 0.25*λ*. Though, sacrifices have been made to obtain these high profiles; by using a *λ*/4-distanced PEC reflector, the antenna bandwidth is restricted by the wavelength-related distance between the PEC reflector and radiator.

To solve the high-profile problem of the PEC reflector antennas, an artificial magnetic conductor (AMC) can be used to replace the PEC reflector [17,18,19,20,21,22]. The AMC provides a 0° reflection phase in a certain frequency and obtains an in-phase reflection characteristic (−90° < *ϕ* < 90°) of the nearby frequencies. Therefore, an AMC reflector can be infinitely close to an antenna if its coupling is not considered. Unfortunately, the AMC reflector has two main drawbacks. (1) The AMC reflector is a periodic structure, and multi-periods are necessary for its function, which may lead to a large reflector size compared to the radiator itself; (2) The AMC is a high-resonance structure with a very high Q-factor that results in narrow bandwidth. Besides, owing to the coupling effect that exchanges electromagnetic energy between the AMC reflector and antenna, it is hard to make the two objects very close to each other; for example, the profile of the design in [23] is 0.16*λ*.

From the above discussion, most of the present unidirectional antennas, include those using a PEC reflector or AMC reflector, are hard to meet all of the characteristics of compact size, low profile, UWB, and unidirectional pattern. However, the 180° reflection phase and the wavelength-related profile characteristics of a PEC reflector make the PEC reflector-based antenna unable to meet the low-profile and UWB characteristics. If the high Q-factor of the AMC at its operating (resonant) frequency results in narrow in-phase reflection bandwidth, then, the AMC reflector-based antennas have the narrow bandwidth characteristic. For wireless applications, such as remote sensing, the characteristics of compact size, low profile, UWB, and unidirectional pattern are required for an antenna. These reasons inspired the idea to design a reflector with a reflection phase much smaller than 180° and to achieve a reflector with large in-phase reflection bandwidth.

To that end, a novel reflector is proposed in this paper. The reflector utilizes the resonant effect of a microwave resonator. It is denoted as a resonance-based reflector (RBR). There are two factors that make the RBR capable of avoiding the trap of narrow bandwidth, which is the problem with the AMC reflector. Firstly, the RBR use open boundaries without a reflect conductor around, thus, its Q factor is usually lower than that of the AMC, so it achieves wider bandwidth. Secondly, the RBR uses the resonance effect of the resonator and operates at a wide frequency band above the resonant frequency. Our theory analysis in Section 2 indicates that, compared to the PEC reflector, the resonance of the RBR would bring ripples to the surface impedance *Z_s_* (the ratio of the electric field intensity on the surface of the conductor to the surface current density) and the reflection phase, and then the reflection phase (*ϕ*) is 0° < *ϕ* < 180° in a very wide frequency band above the resonant frequency. Then, antennas with the characteristics of compact size, low profile, UWB, and unidirectional pattern can be designed by our proposed RBR.

A ring-shaped RBR is proposed in this paper. The ring-shaped RBR presents a wideband reflector for the antenna which could reflect the electromagnetic wave effectively above the resonant frequency. This paper is organized as follows. In Section 2, theory analysis and design of RBR are described. In Section 3, the design process of the ring-shaped RBR-based bowtie antenna (RBR-BA) is discussed; two cases of RBR-BA were designed. The impedance bandwidths of Case 1 and Case 2 are 90% and 78%, respectively, and the front to back ratio (FBR) of Case 1 and Case 2 are 17% and 37%, respectively.

## 2. Analysis and Design of RBR

### 2.1. Theory Analysis of RBR

**Figure 1:**
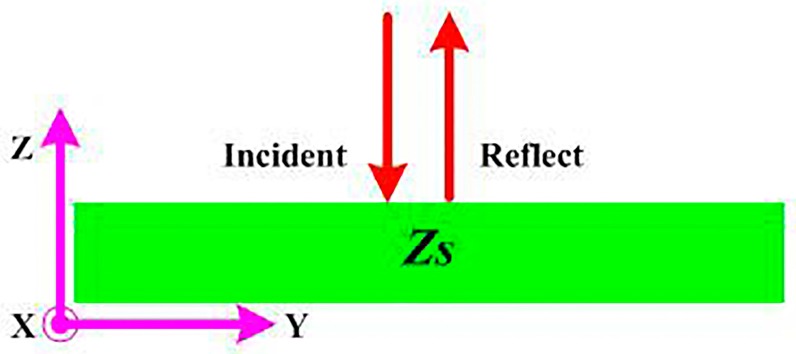
The model of a plane wave incident on the surface.

As shown in Figure 1, a reflector can be considered as surface impedance *Z_s_*. Then, the reflection phase *ϕ* at the surface of the reflector can be defined by Equation (1) [24]. Note that *η* is the characteristic impedance of the free space above the reflector. For Equation (1), the terms “*Im*” and “*ln*” are mathematical symbols. The “*Im*” term means “taking the imaginary part of a complex number”, and the “*ln*” term means “taking the natural logarithm”.

For perfect electric conductor (PEC) and perfect magnetic conductor (PMC), their impedances can be characterized as *Z_s_* ≈ 0 and *Z_s_* ≈ ∞, respectively. Then, a PEC reflector has a reflection phase of 180° and a PMC reflector has a reflection phase of 0°. Therefore, to have constructive interference between the reflecting wave (from the reflector) and the antenna’s direct radiating wave, the two waves should be in phase; then, the distance between the antenna and the reflector should be *λ*/4 for a PEC reflector and should be very small for a PMC reflector. For a PEC reflector, the *λ*/4-distance characteristic also limits the antenna bandwidth. As the PMC does not really exist, the AMC is used as a reflector to realize a very low-profile antenna. However, AMC has the disadvantage of narrow bandwidth. Moreover, the coupling effect causes electromagnetic energy to exchange between the radiator and the AMC reflector, which affects the antenna performances (such as *S* parameter and *Z* parameter), and this is another challenge for antenna design. For these reasons, it is interesting to design a reflector (such as our proposed RBR) with finite surface impedance (0 < *Z_s_* < ∞), so that the reflector achieves a reflection phase *ϕ* range from 0° to 180°.

(1) $$\phi =Im[ln(\frac{Z_{s}-\eta }{Z_{s}+\eta })]$$

**Figure 2:**
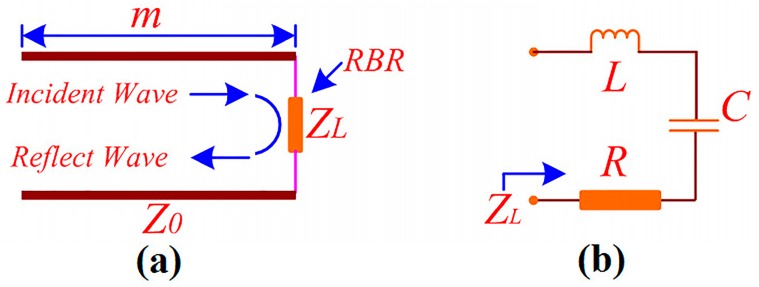
Models of the resonance-based reflector (RBR). (**a**) Transmission line model for the operation; (**b**) the equivalent circuit model.

To depict our proposed RBR, consider the RBR as a loaded impedance *Z_L_* = *R* + *jX* at the terminal of a transmission line, as shown in Figure 2a. *R* is the resistance of the loaded *Z_L_*, and *X* is the reactance of the loaded *Z_L_*. *Z*_0_ is the characteristic impedance of the transmission line (air, in this case, and it is equivalent to *η*), and m is the length of the transmission line (the distance between the RBR and the antenna). Then, the terminal reflection coefficient *Γ_L_* can be defined by Equation (2). Note that *ϕ_L_* is the phase angle of the reflection coefficient *Γ_L_*. As the reflection phase *ϕ* equals *ϕ_L_*, it can be acquired by Equation (3) [25]. In the equation, “*Re*()” means “taking the real part” and “*Im*()” means “taking the imaginary part”.

(2) $$\Gamma_{L}=\frac{Z_{L}-Z_{0}}{Z_{L}+Z_{0}}=\frac{R-Z_{0}+jX}{R+Z_{0}+jX}=\sqrt{\frac{{\left(R-Z_{0}\right)}^{2}+X^{2}}{{\left(R+Z_{0}\right)}^{2}+X^{2}}}e^{j\phi_{L}}$$

(3) $$\phi =\phi_{L}=\arctan(\frac{Im(\Gamma_{L})}{Re(\Gamma_{L})})=\arctan(\frac{2Z_{0}X}{R^{2}-Z_{0}^{2}+X^{2}})$$

From Equation (2), the reflection coefficient *Γ_L_* = −1 = e*^jπ^* for the PEC reflector (*Z_L_* = 0), then, the reflection phase *ϕ* equals 180°. For the PMC reflector (*Z_L_* = ∞), the reflection coefficient *Γ_L_* = 1 = e*^j^*^0^, then, the reflection phase *ϕ* equals 0°. To achieve a reflection phase 0° < *ϕ* < 180°, it is important to realize a *Z_L_* range from 0 to ∞. For this purpose, a resonator may be used as the loaded *Z_L_*.

To further understand the mechanism of the RBR, the resonator can be characterized as an *RLC* (resistance (*R*), inductor (*L*), and capacitor (*C*)) resonator, as depicted in Figure 2b. Then, the loaded *Z_L_* can be calculated by Equation (4). From Equation (4), the impedance of the RBR (*Z_L_*) is finite, and, for the frequencies above the resonance, the value of *Z_L_* increases as angular frequency *ω* (*ω* = 2π*f*) increases. By substituting Equation (4) into Equation (3), the reflection phase can be calculated by Equation (5). From Equation (5), the reflection phase decreases when *ω* increases for the frequencies above the resonance. By substituting Equation (4) into Equation (2), the value of the reflection coefficient can be obtained as Equation (6), and the power above the resonance is reflected.

(4) $$Z_{L}=R+j\frac{\omega^{2}LC-1}{\omega C},|Z_{L}|=\sqrt{R^{2}+X^{2}}=\sqrt{R^{2}+(\frac{\omega^{2}LC-1}{\omega C}}{)}^{2}$$

(5) $$\phi_{L}=\arctan(\frac{2Z_{0}\frac{\omega^{2}LC-1}{\omega C}}{R^{2}+{\left(\frac{\omega^{2}LC-1}{\omega C}\right)}^{2}-Z_{0}^{2}})$$

(6) $$|\Gamma_{L}|\;=\sqrt{\frac{{\left(R-Z_{0}\right)}^{2}+{\left(\frac{\omega^{2}LC-1}{\omega C}\right)}^{2}}{{\left(R+Z_{0}\right)}^{2}+{\left(\frac{\omega^{2}LC-1}{\omega C}\right)}^{2}}}$$

From the above discussion, it is possible to use a resonator as a reflector to achieve constructive radiations with the reflecting wave (from the reflector) and the antenna direct radiating wave in a wide frequency band.

### 2.2. Design of RBR

**Figure 3:**
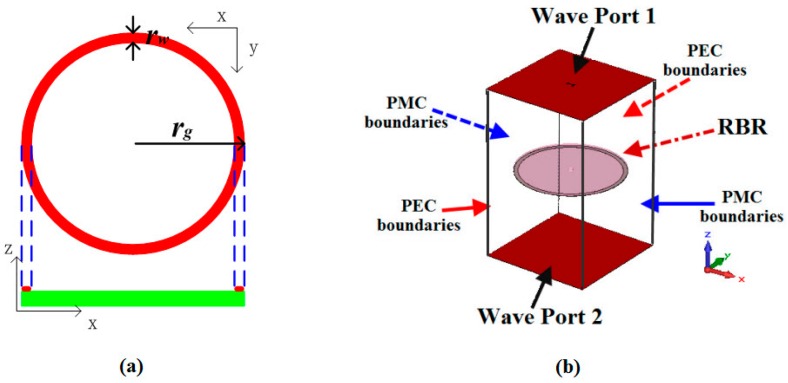
Ring-shaped RBR: (**a**) schematic view; (**b**) simulation model.

A ring-shaped RBR was proposed as shown in Figure 3a, where *r_g_* is the radius of the ring and *r_w_* is the width. The ring is printed on a substrate (named F4B-M and from a local factory) with relative permittivity *ε_r_* = 2.65 and thickness of 1 mm. With the help of a commercial electromagnetic full-wave simulation software named CST Microwave Studio [26], the simulation model utilized to reveal the characteristics of the RBR is depicted in Figure 3b. PMC boundaries were assumed to be at opposite sides, PEC boundaries were assumed to be at the other opposite sizes, and the top and the bottom sides were assumed to be wave ports that imitate infinitely long waveguides for excitation and absorption.

**Figure 4:**
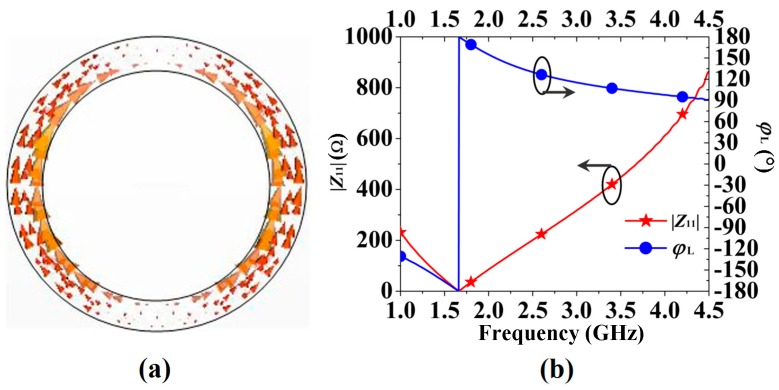
Characteristics of the RBR: (**a**) the surface current of the proposed RBR; (**b**) impedance and phase characteristics of the proposed RBR.

Assigning *r_g_* = 25.8 mm and *r_w_* = 2 mm, then, the surface current distributions at the resonant frequency *f_r_* = 1.72 GHz of the ring were plotted, as shown in Figure 4a. As shown in the figure, the currents originate from the bottom of the ring and flow along the two half-rings to the top. It is interesting to find that the perimeter of the central line of the ring (2 × π × (*r_g_* − *r_w_*/2) = 155.8 mm) is almost equal to the wavelength of the resonance *f_r_* (*λ_r_* = 174.4 mm). Then, if the ring RBR is designed to other frequencies, we can firstly determine its perimeter as a wavelength *λ*. From the current distribution shown in Figure 4a, the currents originate from the bottom and flow along two half-rings to the top, therefore, the ring operates as two *λ*/2 resonators. Note that the effective length of the resonators is longer than a half-ring, as the two half-rings are connected.

The |*Z*_11_| (*Z*_11_ is the input impedance of the wave port 1) curve and the reflection phase (*ϕ*) curve of the ring-shaped RBR are shown in Figure 4b. From the red curve, |*Z*_11_| equals 0 at *f_r_* = 1.72 GHz and it is almost growing linearly with increasing frequency. Then, *ϕ* of the RBR is 180° at *f_r_* = 1.72 GHz, and decreases to 90° with a frequency increase to 4.5 GHz. These characteristics conform to Equations (4) and (5). We must point that the resistance *R* of the equations represents the power pass through the resonator (reflector); therefore, *R* is a function of frequency and equals zero at *f_r_* = 1.72 GHz.

**Figure 5:**
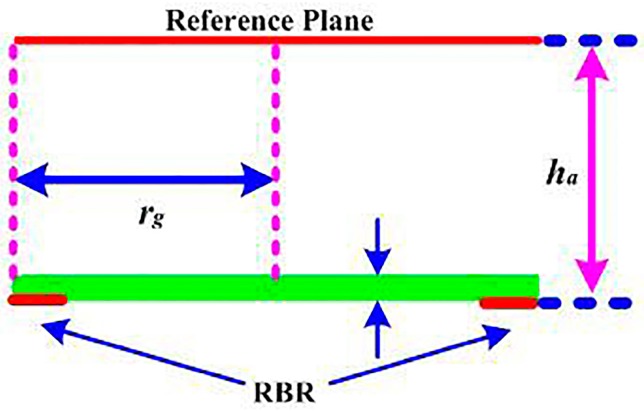
The schematic view of the reference plane.

From the above discussion, the ring-shaped RBR achieves reflected phase *ϕ* < 180° in a very wide frequency band. However, in-phase reflection requires −90° < *ϕ* < 90°. To this end, a radiator must be placed at a certain distance (*h_a_*) from the RBR to introduce a space phase (2*βh_a_* = 4π*h_a_*/*λ*, where *β* is the phase constant of the free space). The distance *h_a_* can also be obtained from full-wave simulation, and, as shown in Figure 5, the radiator is placed at the reference plane with distance *h_a_* = 14 mm to the RBR. Then, the reflection phase *ϕ* at the RBR surface is denoted as *ϕ*_1_, and the reflection phase *ϕ* at the reference plane is denoted as *ϕ*_2_.

**Figure 6:**
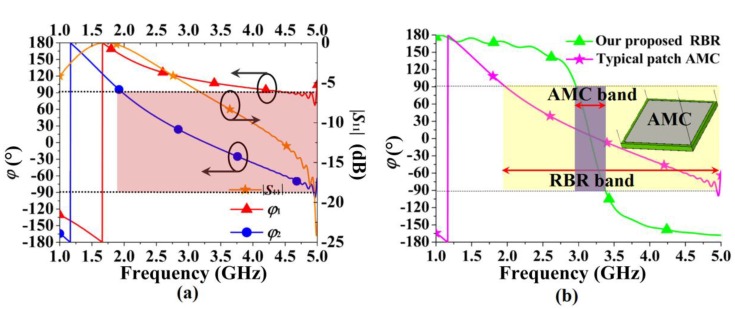
Reflection phases: (**a**) reflection phase *ϕ* and |*S*_11_| of RBR; (**b**) comparison of the in-phase band for the typical patch artificial magnetic conductor (AMC) and the proposed RBR.

The *ϕ*_1_ and *ϕ*_2_ curves were plotted (see Figure 6a) for comparison, and the curve of the magnitude of the scattering parameter (|*S*_11_|) is also shown in the figure. The reflection phase *ϕ*_2_ at the reference plane is smaller than *ϕ*_1_, as expected, and, *ϕ*_2_ achieves an in-phase reflection band (−90° < *ϕ* < 90°) from 1.97 GHz to 4.94 GHz (86%). Though, the reflection coefficient |*S*_11_| decreases as frequency increases, and approving reflection is obtained for the frequencies close to that of the resonance. To present the merit of the proposed RBR, Figure 6b compares *ϕ*_2_ of the proposed RBR with the reflection phase of a typical square AMC. The AMC has an element size of 25 mm × 25 mm, and the patch is printed on a substrate with *ε_r_* = 2.2 and thickness of 2 mm. As shown in Figure 6b, the in-phase reflection band of the AMC is 3.16–3.60 GHz (13%). Thus, the proposed RBR demonstrates a much wider in-phase reflection band than the AMC.

**Figure 7:**
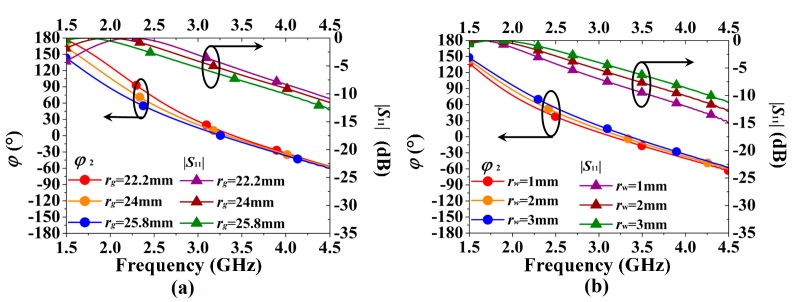
Parametric study: (**a**) radius *r_g_* and (**b**) width *r_w_* of the ring-shaped RBR.

To further investigate the ring-shaped RBR, parametric studies were conducted, as exhibited in Figure 7. The two parameters *r_g_* and *r_w_* were swept in with their results, plotted in Figure 7a,b, respectively. It can be inferred from Figure 7a that the resonance of the RBR increases as *r_g_* decreases, and the resonant frequency increases from 1.72 GHz to 1.98 GHz and 2.23 GHz when *r_g_* decreases from 25.8 mm to 24 mm and 22.2 mm. Therefore, the parameter *r_g_* is useful for operational frequency tuning. From Figure 7b, the width *r_w_* of the RBR has more impact on |*S*_11_| than on *ϕ*_2_. The resonant frequency increases from 1.62 GHz to 1.72 GHz and 1.82 GHz when *r_w_* increases from 1 mm to 2 mm and 3 mm, while the |*S*_11_| increases about 1.2 dB when *r_w_* increases by 1 mm. Meanwhile, the in-phase reflection band (−90° < *ϕ* < 90°) is little affected by *r_w_*, as the bandwidths for *r_w_* = 1 mm, 2 mm, and 3 mm are 1.87–4.82 GHz, 1.97–4.94 GHz, and 2.06–5.09 GHz, respectively. Thus, larger *r_w_* means better FBR when the RBR is used as reflector for an antenna.

## 3. Antenna Design and Discussion

### 3.1. Antenna Design

**Figure 8:**
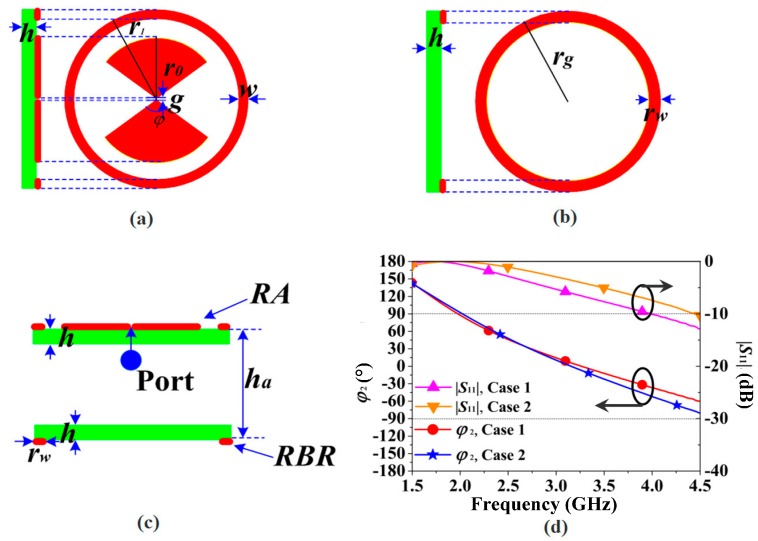
The design of the RBR-bowtie antenna (RBR-BA): (**a**) schematic view of reference antenna (RA); (**b**) schematic view of RBR; (**c**) side view of RBR-BA; (**d**) the characteristics of the RBRs.

The proposed ring-shaped RBR was used to design a unidirectional antenna, as shown in Figure 8. The schematic view of the reference antenna (RA) and the ring-shaped RBR are demonstrated in Figure 8a,b, respectively. The RA has an identical structure to the RAs of our previous proposed antennas [13,27]. The RA is a bowtie antenna surrounded by a metal ring. Therefore, it is a UWB antenna with an omnidirectional pattern. From simulations, the antenna parameters were obtained. The parameters of the RA are *r*_1_ = 26 mm, *r*_0_ = 20 mm, w = 0.8 mm, *g* = *h* = 1 mm, and *ϕ* = 120°. To obtain a unidirectional pattern, the RBR is used with the RA, as exhibited in Figure 8c. Then, the proposed RBR-bowtie antenna (RBR-BA) was constructed. From the above discussion of Figure 7, the width *r_w_* has big impact on FBR. Therefore, two cases of RBR-BA with different RBR sizes were designed for comparison. The RBR sizes of the two cases are demonstrated in Table 1. Case 1 used the previously designed RBR size, and Case 2 utilized an RBR with a larger ring width *r_w_*. The characteristics of the RBRs of the two cases were plotted and are shown in Figure 8d. As shown in the Figure 8d, the in-phase reflection *ϕ*_2_ band of Case 2 is a little narrower than Case 1, while the reflection coefficient |*S*_11_| band of Case 2 is much larger Case 1. Then, it can be expected that the antenna using Case 2 RBR would have a better FBR than the antenna using Case 1 RBR.

**Figure 9:**
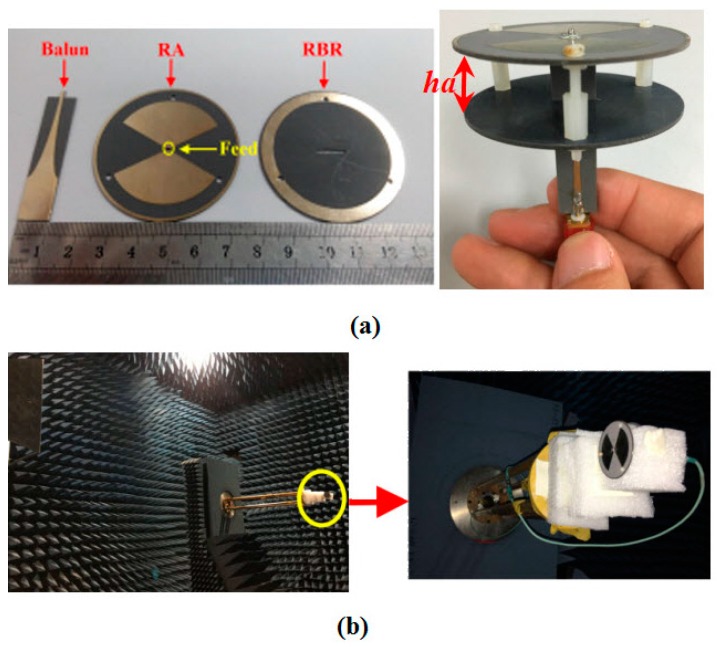
Pictures of (**a**) top view and side view of the balun, RA, RBR, and RBR-BA; (**b**) measurement environment.

**Figure 10:**
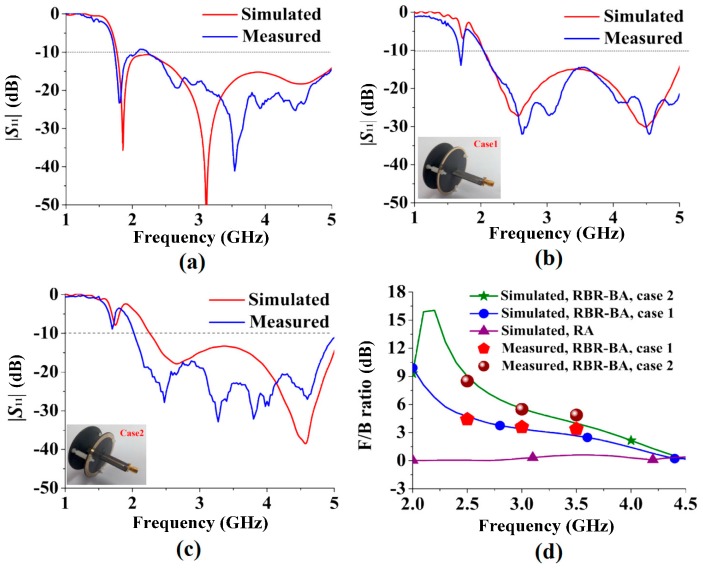
Results of the antennas: (**a**) |*S*_11_| curves of the RA; (**b**) |*S*_11_| curves of the RBR-BA Case 1; (**c**) |*S*_11_| curves of the RBR-BA Case 2; (**d**) front-to-back ratio (FBR) curves of the RA and the RBR-BA Case 1 and Case 2.

The antennas were manufactured and measured as shown in Figure 9a,b. Similar to [13], a wide-band balun was used to transform the 75 Ω input impedances of the antennas to 50 Ω of a subminiature version A (SMA) connector for measurement. The |*S*_11_|was measured by an Agilent N5230A network analyzer, and the radiation patterns were measured in a microwave chamber with NSI2000 system. The Agilent N5230A network analyzer is an *S*-parameter measuring instrument from Agilent Ltd. (Santa Clara, CA, USA) and the NSI2000 system is a field measuring system from NSI Ltd. (Torrance, CA, USA). The simulated and measured |*S*_11_| curves of the RA and the RBR-BA Case 1 and Case 2 are plotted in Figure 10a–c, respectively. The high cutoff frequencies of all the simulated and measured results of the antennas are over 5 GHz. The simulated impedance bandwidths are 1.76–5.11 GHz (97.4%), 2.04–5.14 GHz (86.3%), and 2.16–5.14 GHz (81.6%) for the RA and the RBR-BA Case 1 and Case 2, respectively, while the measured results are 1.74–5.12 GHz (98.5%) for the RA, 2.04–5.12 GHz (86.3%) for Case 1, and 1.97–5.01 GHz (87.1%) for Case 2. The simulated and measured FBR are pictured in Figure 10d. Notice that, as the reflection coefficient of the RBR (Figure 6a) makes the FBR of the antenna nearly 0 dB for the frequencies above 4.5 GHz (bidirectional patterns, as with the RA); the FBR values above 4.5 GHz are not shown here. To verify the simulated FBR results, FBR values for frequencies of 2.5 GHz, 3 GHz, and 3.5 GHz were measured. For 2.5 GHz, 3 GHz, and 3.5 GHz, the simulated/measured FBRs of Case 1 are 4.73/4.42 dB, 3.39/3.58 dB, and 2.64/3.38 dB, respectively. For 2.5 GHz, 3 GHz, and 3.5 GHz, the simulated/measured FBRs of Case 2 are 9.00/8.50 dB, 5.56/5.49 dB, and 3.98/4.87 dB, respectively. The differences between the simulated and measured results are small (ranging from 0.07 dB to 0.89 dB). Therefore, the measured results agree with the simulated results. From Figure 10d, the omnidirectional pattern of RA (with FBR = 0 dB) is transformed to unidirectional of the RBR-BA. Moreover, Case 2 shows better FBR than Case 1, as predicted. The measured FBR of Case 1 at 2.5 GHz, 3 GHz, and 3.5 GHz are 4.42 dB, 3.58 dB, and 3.38 dB, respectively, while the measured FBRs of Case 2 for 2.5 GHz, 3 GHz, and 3.5 GHz are 8.5 dB, 5.49 dB, and 4.87 dB, respectively.

In Table 2, our works are compared to some previous studies in [7,15,21,22]. Compared to [7,15], our works show advantages of compact size, low profile, and wide bandwidth. Compared to [21], our works realize a much wider bandwidth. Furthermore, our works demonstrate a smaller size and wider bandwidth in comparison to [22]. Therefore, our proposed design methodology is valuable for antenna design with compact, low-profile, wide band, and unidirectional pattern characteristics.

**Figure 11:**
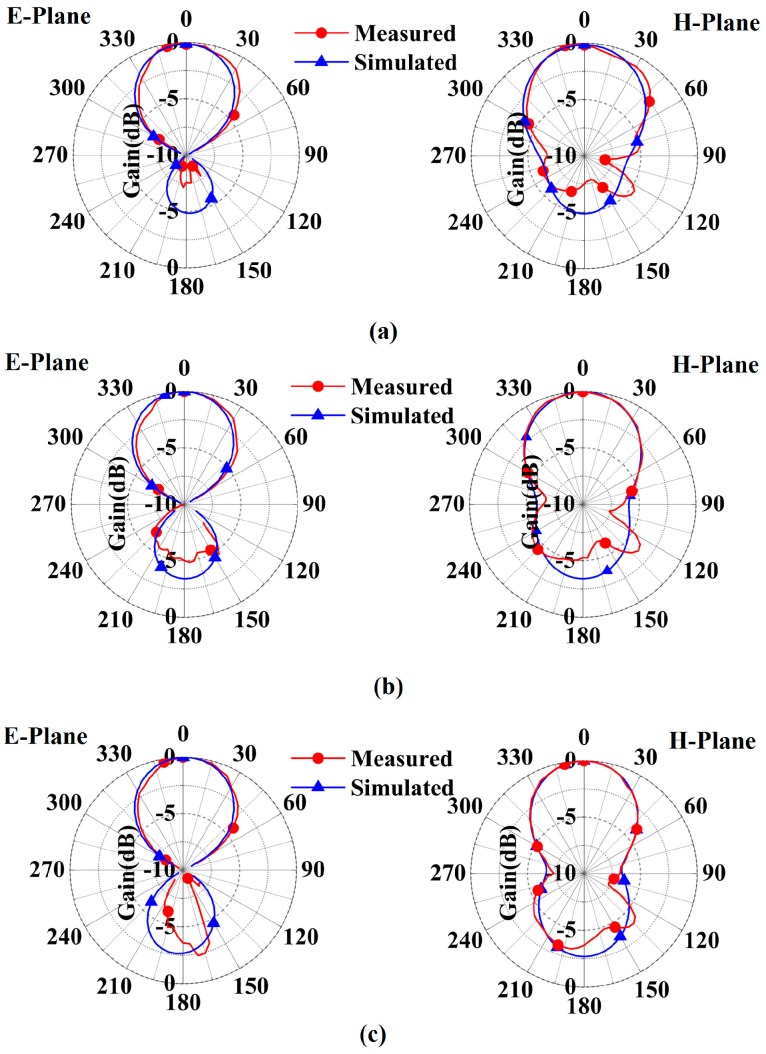
Measured and simulated patterns of the RBR-BA Case 1: (**a**) 2.5 GHz; (**b**) 3.0 GHz; (**c**) 3.5 GHz.

**Figure 12:**
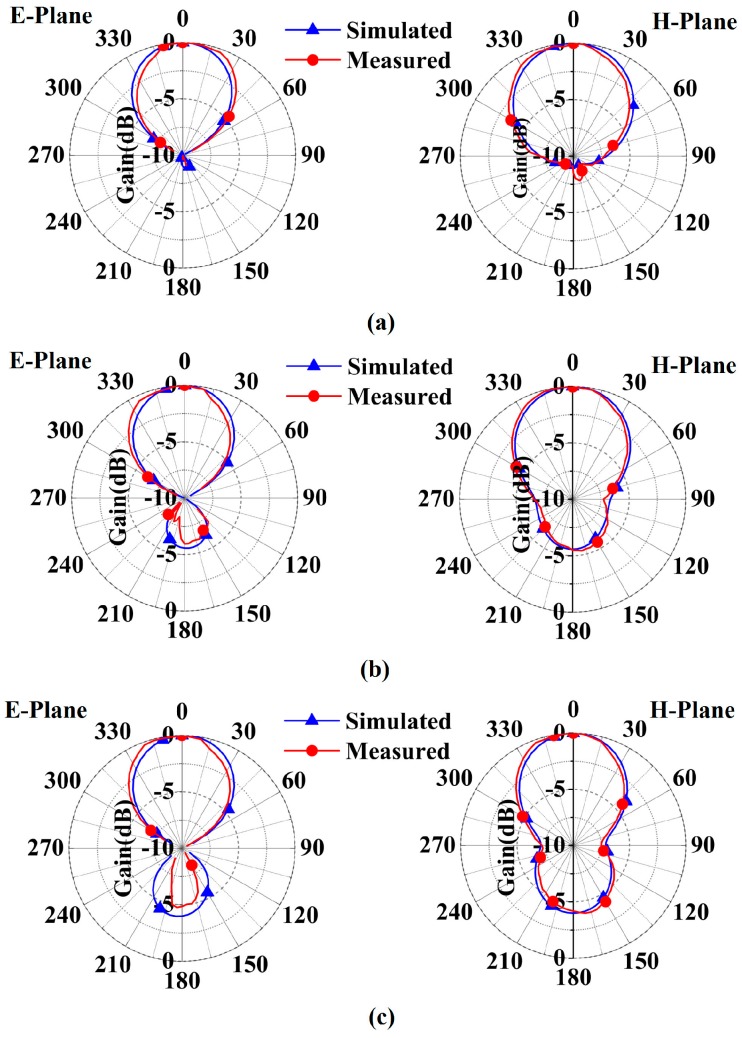
Measured and simulated patterns of the RBR-BA Case 2: (**a**) 2.5 GHz; (**b**) 3.0 GHz; (**c**) 3.5 GHz.

The measured and simulated patterns of the RBR-BA Case 1 are demonstrated in Figure 11. The patterns of the three frequencies of 2.5 GHz, 3.0 GHz, and 3.5 GHz are depicted in Figure 11a–c, respectively. Figure 12a–c shows the measured and simulated patterns of 2.5 GHz, 3.0 GHz, and 3.5 GHz of the RBR-BA Case 2, respectively. Note that the E plane is a plane parallel to the maximal electric field vector, and the H plane is a plane parallel to the maximal magnetic field vector. As shown in the figures, the measured main lobes (front radiation) are very similar to their simulated ones, while the measured side lobes (back radiation) show discrepancies to the simulated results. The discrepancies between the simulations and measurements in the back radiations may be caused by the balun, which would affect the back radiation slightly. However, the measured results still confirm unidirectional radiations of these frequencies.

### 3.2. Discussion

From Figure 8, the radius *r_g_* and width *r_w_* of the rings for the RBR are important for the RBR characteristics of the reflection coefficient and in-phase reflection band. In this section, the impacts of the *r_g_* and *r_w_* on the RBR-BA performances are investigated. For simplicity, only the RBR-BA Case 1 was used for parametric study. This means when *r_w_* or *r_g_* was changed, the other antenna parameters were fixed and identical to Case 1.

**Figure 13:**
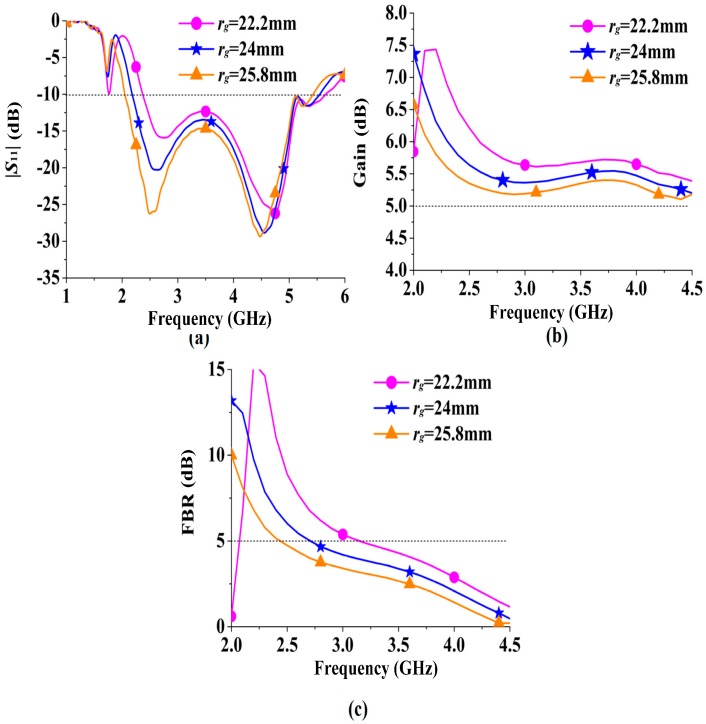
Parametric study in terms of *r_g_*: (**a**) scattering parameter S_11_; (**b**) gain; (**c**) FBR.

In Figure 13, the effects of *r_g_* on the antenna performances are presented. It can be inferred from Figure 13a that decreasing *r_g_* leads to increasing of the low cutoff frequency of the antenna. As *r_g_* decreased from 25.8 mm to 24 mm and 22.2 mm, the −10 dB low cutoff frequency increased from 2.04 GHz to 2.18 GHz and 2.37 GHz. Meanwhile, with the decreasing of the circumference of the ring-shaped RBR, the resonance frequency will increase, which makes the summits of the peak gain and the FBR move to higher frequencies, as depicted in Figure 13b,c.

**Figure 14:**
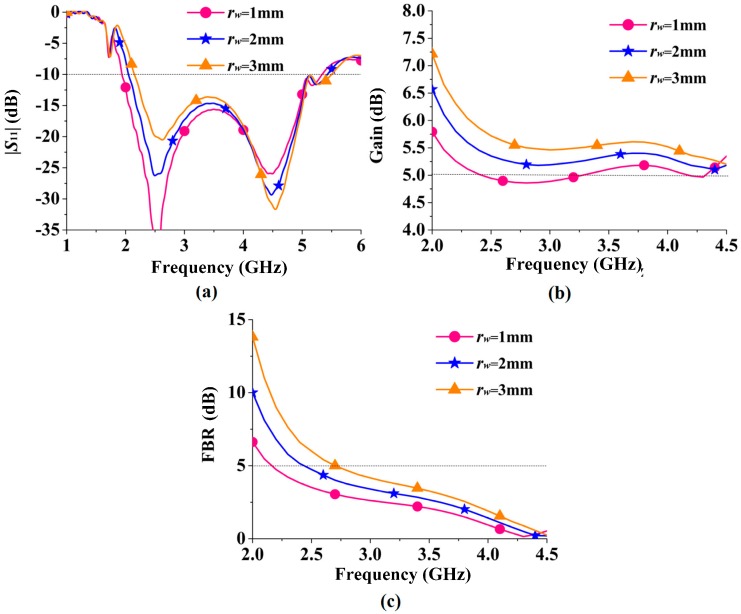
Parametric study in terms of *r_w_*: (**a**) S_11_; (**b**) gain; (**c**) FBR.

As shown in Figure 14a, the −10 dB low cutoff frequency increases from 1.94 GHz to 2.04 GHz and 2.15 GHz when *r_w_* increases from 1 mm to 2 mm and 3 mm. As shown in Figure 14a, *r_w_* has a small effect on the |*S*_11_| curve. However, from Figure 14b,c, it is important for the gain and the FBR enhancements. From Figure 14b, the gain of the proposed RBR-BA increases with increasing *r_w_*, typically 0.5 dB with a 1 mm increment of *r_w_*. In Figure 14c, the same trend happens to the FBR, as typically a 1.5–2.8 dB increment is obtained with a 1 mm increase of *r_w_*. These characteristics are in accord with the discussions of Figure 7b.

## 4. Conclusions

A novel reflector of a resonance-based reflector (RBR) was proposed in this research. The concept and theory of the RBR were discussed. A ring-shaped RBR was investigated and used to design unidirectional antennas. A novel broadband unidirectional antenna that utilizes the RBR was proposed in this paper. Compared to the PEC and the AMC reflectors, the proposed RBR is capable of realizing an antenna with advantages of low profile, compact size, ultrawideband and unidirectional pattern, simultaneously. Two cases of RBR-BA were designed, and their measured results agreed with simulation results. This research indicates that the proposed RBR is a good choice for antenna design with low-profile, compact size, ultrawideband and unidirectional pattern characteristics, which makes it a good candidates for wireless applications such as indoors communication, remote sensing, and wireless sensor systems.

## Figures and Tables

**Table 1:** The parameters of RBR-BA.

	Para.	*r_g_* (mm)	*r_w_* (mm)	*h_a_* (mm)
Ant.				
**Case 1**		25.8	2	14
**Case 2**		26	4.3	16.3

**Table 2:** Comparison of our work and previous studies.

Reference	Area (mm^2^)	Profile (mm)		Impedance Bandwidth (%)
**[7]**	1.47*λ* × 3.77*λ*	0.27*λ*		31.8%
**[15]**	2.05*λ* × 2.05*λ*	0.29*λ*		20%
**[21]**	0.48*λ* × 0.48*λ*	0.01*λ*		18%
**[22]**	1.13*λ* × 1.13*λ*	0.05*λ*		25.4%
**Our work**	0.33*λ* × 0.33*λ*	Case 1	0.09*λ*	86.3%
		Case 2	0.11*λ*	87.1%
